# Particle Beam Radiation Therapy for Adenoid Cystic Carcinoma of the Nasal Cavity and Paranasal Sinuses

**DOI:** 10.3389/fonc.2020.572493

**Published:** 2020-09-30

**Authors:** Weixu Hu, Jiyi Hu, Qingting Huang, Jing Gao, Jing Yang, Xianxin Qiu, Lin Kong, Jiade J. Lu

**Affiliations:** ^1^Department of Radiation Oncology, Shanghai Proton and Heavy Ion Center, Shanghai, China; ^2^Shanghai Engineering Research Center of Proton and Heavy Ion Radiation Therapy, Shanghai, China; ^3^Department of Radiation Oncology, Shanghai Proton and Heavy Ion Center, Fudan University Cancer Hospital, Shanghai, China

**Keywords:** nasal cavity and paranasal sinuses adenoid cystic carcinoma, radiotherapy, particle-beam radiation therapy, proton beam therapy, carbon-ion radiotherapy

## Abstract

**Background:** Sinonasal adenoid cystic carcinoma (SNACC) presents a challenge to oncologists due to its complex anatomy and poor prognosis. Although radiation therapy, either definitive or adjuvant to surgery, is an important part of the multidisciplinary management of SNACC, photon-based radiotherapy yielded suboptimal local control. The purpose of this study was to report the clinical results of a large patient cohort treated with particle beam radiation therapy.

**Methods:** Patients with SNACC that received proton beam therapy (PBT), carbon-ion radiotherapy (CIRT) or a combination of CIRT and PBT between May 2015 and May 2019 were included in the analysis. Three patients were treated with PBT, 17 with CIRT and 18 received PBT and a CIRT boost. Overall survival (OS), progression-free survival (PFS), local control (LC), regional control (RC), and distant metastasis-free (DMF) rates were calculated using the Kaplan-Meier method. Toxicities were reported using the CTCAE (version 4.03).

**Results:** A total of 38 patients were included in this analysis. Of these patients, 12 had recurrent disease, including 10 whose previous photon-based RT had failed. The most common primary tumor site was the maxillary sinus. Thirty-six patients (94.7%) suffered from locally advanced disease (T3-4). After a median follow-up of 27.2 months, the 3-year OS, PFS, LC, RC, and DMF rates were 96.7, 80.6, 90.0, 100, and 88.7%, respectively. No acute toxicities of grade 3 or above were observed. Two patients experienced grade 3 xerostomia or vision decreased, and one patient died of hemorrhage.

**Conclusion:** PBT, CIRT or a combination of CIRT and PBT appeared to be a promising treatment option for SNACC and produced satisfactory local control and toxicity profile. Longer follow-up is needed to verify the long-term benefit of particle-beam radiation therapy (PBRT) for patients with SNACC.

## Introduction

Adenoid cystic carcinoma (ACC) is a rare condition that accounts for 3–5% of all head and neck malignancies (1). ACC is characterized by a slow growth rate but a high probability of local infiltration, perineural spread, and distant metastasis. ACC usually arises in the major salivary glands; however, with 10–25% of ACC cases originating in the nasal cavity and paranasal sinuses, it is responsible for 5–15% of sinonasal malignancies (2). Sinonasal ACC (SNACC) presents a challenge to oncologists due to its complex anatomy and poor prognosis compared with carcinomas that affect the major salivary glands. The 5-year overall survival (OS) rate is approximately 60% for patients with SNACC compared with 90% for all head and neck ACC (3).

Although surgery is the mainstay of treatment for head and neck ACC, the 10-year local control (LC) of surgery alone was only 60%, and even as low as 30% in patients with positive margins (4). For SNACC patients, complete resection within sufficient safety margins is usually not feasible. Therefore, radiation therapy (RT), either definitive or adjuvant to surgery, is an important part of the multidisciplinary management of SNACC. However, ACC is resistant to photon-based RT (5). This is part of the reason why SNACC, a condition that is also not amenable to surgical resection, has a poor prognosis with the 5-year DSS rate being only 37.3% when treated with RT alone (6). It is clear that a more effective local treatment is needed.

Particle-beam radiation therapy (PBRT), which uses protons or heavier ions such as helium or carbon ions, is relevant in the treatment of SNACC. A particle beam deposits low doses of radiation along its travel path through the body, before reaching its target and distributing most of its dose immediately prior to termination at the Bragg peak. The lateral penumbra of particle beams is significantly sharper than that in photon beams. Compared with photon-based intensity-modulated RT (IMRT), this feature of PBRT improves the therapeutic ratio by introducing a sharp dose gradient between the tumor volume of and adjacent to critical organs at risk (OARs). Also, high linear energy transfer (LET) particles, such as carbon ions, have a relative higher biological effectiveness (RBE) of 2–5, depending on beam energy, fraction dose, and the cell and tissue types irradiated, as compared to photons and protons (the prescribed proton doses used RBE value of 1.1) (7, 8). Both features are of especially importance in the management of SNACC for overcoming the condition's anatomical complexity and resistance to conventional RT. However, while there is ample evidence describing the use of PBRT in heterogeneous ACC patient populations, there is minimal specific data available in relation to SNACC.

The purpose of this study was to report the clinical results of a large patient cohort treated with PBRT at the Shanghai Proton and Heavy Ion Center (SPHIC) over the past five years, including local control, survival and adverse events associated with PBRT.

## Methods

### Pre-treatment Workups

All newly diagnosed cases were confirmed by pathology. For patients with recurrent disease, pathological and/or radiological diagnoses were required. All patients were evaluated according to the standardized pre-radiation work-ups of the SPHIC for head and neck cancers. This included a complete history and physical examination (H&P); imaging study of the head and neck region (enhanced magnetic resonance imaging [MRI] was preferred, but computerized tomography [CT] with contrast was permitted if MRI was contraindicated or declined by the patient); routine lab tests (full blood count; serum electrolytes; liver and kidney function tests); fluorodeoxyglucose-positron emission tomography/CT (FDG-PET/CT) (or a thoracic/abdominal CT and a whole body bone scan); urine analysis; and an electrocardiogram (EKG). All disease was staged according to the seventh (diagnosed before 1 January, 2018) or eighth (diagnosed after 1 January, 2018) edition of the AJCC (American Joint Committee on Cancer) TNM (tumor, nodes, metastases) staging system. This study was approved by SPHIC's institutional review board (IRB) with a waiver of informed consent.

### Particle Beam Radiation Therapy

AlphaCradle® and thermoplastic masks were used to immobilize patients in a supine position. Simulation CT of the head and neck region without intravenous (IV) contrast was performed at a slice thickness of 1.5 mm. Simulation MRI was also carried out, and then MRI-CT fusion was used to delineate the target and OARs. All disease foci discovered on clinical examination or during imaging studies were the gross tumor volumes (GTVs). Three clinical target volumes (CTVs) were delineated: CTV-G or CTV-N covered primary site (GTVp) or positive neck lymph nodes (GTVnd) plus 1–3 mm depend on the surrounding OARs. CTV 1 included the tumor bed (after R1 resection), the pretreatment tumor bed (the tumor region before R2 resection) and high-risk areas for tumor extension; CTV 2 included the ipsilateral or bilateral jugular lymph node region, depending on cervical lymph node status and primary tumor extension Planning target volumes (PTVs) included the CTVs plus a 3–6 mm expansion to account for range uncertainty and setup errors. OARs delineated included the brain, temporal lobes, brainstem, spinal cord, optic nerves and chiasm, lenses, cochleae, parotid glands, and larynx. Doses of intensity-modulated proton or carbon-ion therapy (IMPT or IMCT) were prescribed in Gy (RBE). The RBE value for proton radiotherapy was 1.1 and for carbon-ion radiotherapy was between 2.8 and 3.7 (depending on the depth in the spread-out Bragg peaks). The dose constraints for OARs were based on normal tissue tolerance as described by Emami et al. (9) and the National Institute of Radiation and Quantum Science (QST Hospital) (10). Intensity-modulated proton therapy (IMPT) and intensity-modulated carbon-ion therapy (IMCT) were delivered with pencil-beam scanning (PBS). Multi-field optimization (MFO) with two to three arrangements was used in most treatment plans. The Siemens Syngo treatment planning system (TPS) was used to plan the IMPT and IMCT. CT without IV contrast was performed weekly on all patients to verify the dose distribution.

### Systemic Therapy

No patient in this cohort received concurrent systemic therapy, such as chemotherapy or target therapy, during PBRT. Eleven patients received induction chemotherapy or target therapy. The most commonly used chemotherapy regimens were adriamycin-cyclophosphamide-cisplatin, gemcitabine-cisplatin, or apatinib.

### Follow-Up and Toxicity Evaluation

Patients were treated as in-patients and evaluated for acute toxicities daily. Weekly CT scans started from week 2 of PBRT and were used to evaluate response to treatment and any need to re-plan PBRT due to substantial anatomical alterations. Following the completion of treatment, patients were evaluated for adverse effects and disease control according to SPHIC's standardized institutional follow-up protocol. The first follow-up was at four weeks after PBRT completion. The patients were then followed up every three to four months for two years, every six months for three more years, and yearly thereafter.

Each follow-up included a complete H&P, routine lab tests, and imaging studies of the head and neck regions. Other tests, such as PET-CT scans, were also carried out, depending on clinical findings at the time of follow-up. Acute toxicities were defined as those that occurred within 3 months at the initiation of PBRT. Toxicities that occurred after this period until the last follow-up were late toxicities. Both acute and late toxicities was evaluated by the Common Terminology Criteria for Adverse Events (CTCAE) version 4.03.

### Statistical Analysis

OS was defined as the duration between diagnosis and death or last follow-up. Local control (LC), regional control (RC), and distant metastasis-free (DMF) were defined as the duration between diagnosis and corresponding failure of control. The LC, RC, DMF, progression-free survival (PFS), and OS rates were calculated using the Kaplan-Meier method performed with SPSS (Version 25.0). Univariate analysis using COX regression model performed with SPSS (Version 25.0). Univariate analysis using competing risk model performed with R statistical software (version3.4.1; R Foundation, Austria).

## Results

### Patient Characteristics

Forty-one consecutive patients with SNACC were treated at SPHIC between May, 2015, and May, 2019. Three of these patients had distant metastasis (DM) at diagnosis and were excluded. The remaining 38 were included in the analysis. Of these patients, 12 had recurrent disease, including 10 whose previous photon-based RT had failed. The most common primary tumor site was the maxillary sinus. Only two patients presented with T2 disease. The remaining patients suffered from locally advanced disease (T3-4), including one patient with bilateral neck adenopathy. Twenty-nine patients (76.3%) had skull base involvement. Due to extension of the primary tumor, R0 or R1 resections were only achieved in six patients. Thirty-two patients (84.2%) had gross residual tumor before receiving PBRT, and the median GTV volume was 56.8 ml. Eleven patients received induction chemotherapy or target therapy before PBRT. The patients' characteristics are detailed in Table 1.

**Table 1:** Characteristics of patients.

**Characteristics**	**No. (%)**
**Total**	**38 (100%)**
**Gender**
Male	18 (47.4%)
Female	20 (52.6%)
**Age**
Median(range), years	45 (17–75)
**T category**	
T1	0
T2	2 (5.3%)
T3	7 (18.4%)
T4	29 (76.3%)
**N category**	
N0	37 (97.4%)
N2	1 (2.6%)
**Tumor location**	
Maxillary sinus	30 (78.9%)
Ethmoid sinus	4 (10.5%)
Nasal cavity	4 (10.5%)
**Skull base involvement**	
Yes	29 (76.3%)
No	9 (23.7%)
**Surgery**	
Without surgery	4 (10.5%)
With surgery	23 (60.5%)
Biopsy	11 (28.9%)
**Gross tumor**	
With gross tumor	32 (84.2%)
Without gross tumor	6 (15.8%)
**GTV** ml, median (range)	56.8 (7.7–183.6)
**Re-irradiation**	
Irradiation naïve	28 (73.7%)
Re-irradiation	10 (26.3%)
**Disease status**	
Initial disease	26 (68.4%)
Recurrent disease	12 (31.6%)
**Radiation technique**	
Proton	3 (7.9%)
CIRT	17 (44.7%)
Proton and CIRT	18 (47.4%)
**Median dose to GTV (range), Gy (RBE)**	69.5 (56–73.5)
**Induction chemotherapy or apatinib therapy**	
Yes	11 (28.9%)
No	27 (71.1%)

### Particle-Beam Radiotherapy

Three patients were treated with proton beam therapy (PBT), 17 with carbon-ion radiotherapy (CIRT) and 18 received PBT and a CIRT boost.

The patients who had R0 resections received PBT using 56 Gy (RBE) in 28 fractions at the surgical bed and high-risk regions. Of the 4 patients who had R1 resections, 3 received CIRT using 63 Gy (RBE) in 18 fractions to the tumor bed and 54 Gy (RBE) in 18 fractions to the high risk regions using the simultaneous integrated boost technique. The other R1 patient received PBT (56 Gy (RBE)/28 fractions) followed by a CIRT boost (15 Gy (RBE)/5 fractions). Of the patients with gross disease at their initial treatment, 1 received PBT (66 Gy (RBE)/30 fractions), 4 received CIRT (63-70 Gy (RBE) at the gross tumors and 54-60 Gy (RBE) at the high-risk regions in 18-20 fractions), and 17 patients received a combination of PBT (56 Gy (RBE)/28 fractions) and a CIRT boost (15-17.5 Gy (RBE)/5 fractions). Based on cervical lymph node status as well asand the location and extension of the primary tumor, unilateral neck irradiation was performed in nine patients, and bilateral neck irradiation was performed in seven patients.

**Figure 1:**
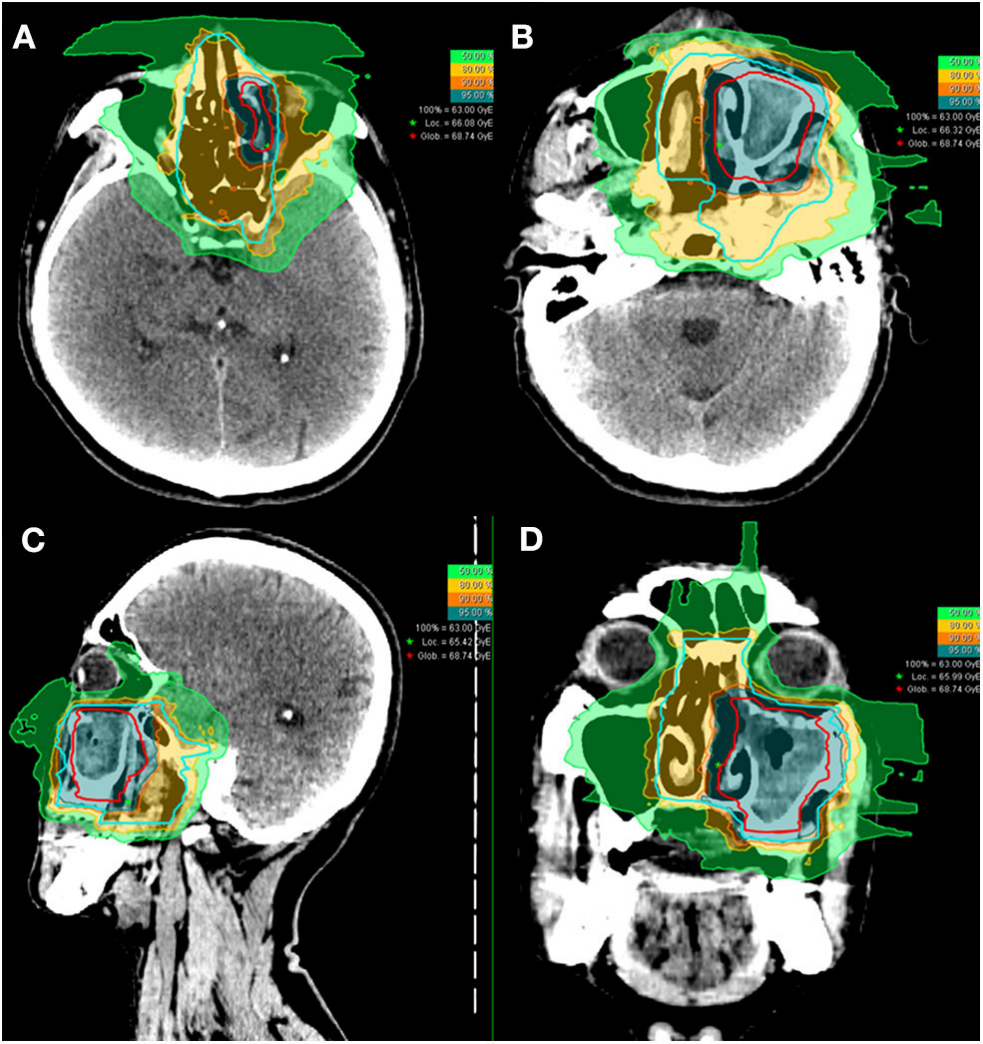
Axial **(A,B)**, sagittal **(C)**, and coronal **(D)** views of a typical intensity-modulated carbon ion radiotherapy treatment plan.

**Figure 2:**
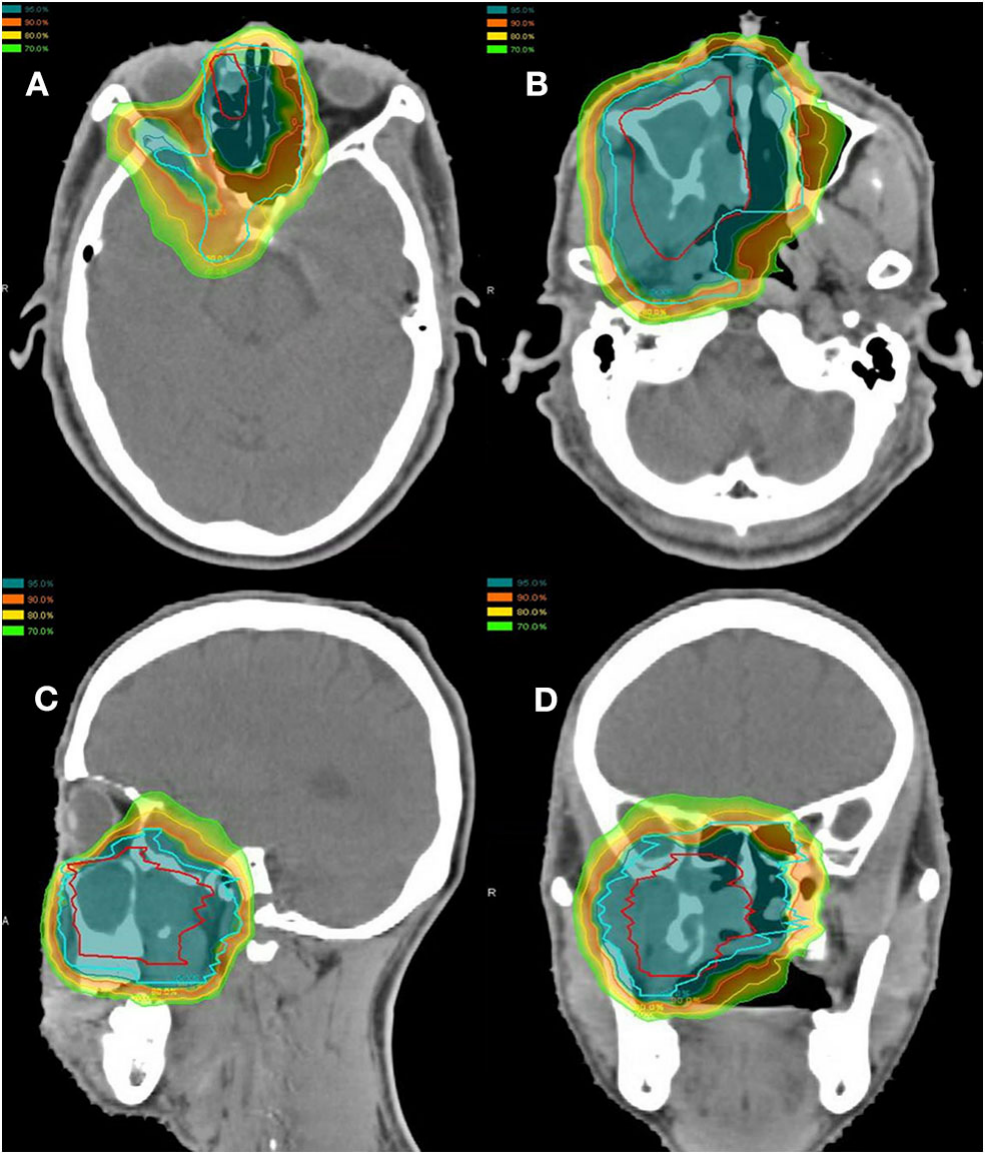
Axial **(A,B)**, sagittal **(C)**, and coronal **(D)** views of a typical intensity-modulated proton and carbon ion-boost radiotherapy treatment plan.

Of the 10 patients who were re-irradiated, CIRT using 60-66 Gy (RBE) in 20-22 fractions was given to the recurrent tumor and 54-59.4 Gy (RBE) in 20-22 fractions for the high-risk regions. None of the re-irradiated patients received CIRT to the neck. Typical treatment plans for the combination of PBT and CIRT boost or CIRT alone are illustrated in Figures 1, 2.

### Disease Control and Survival Outcomes

**Figure 3:**
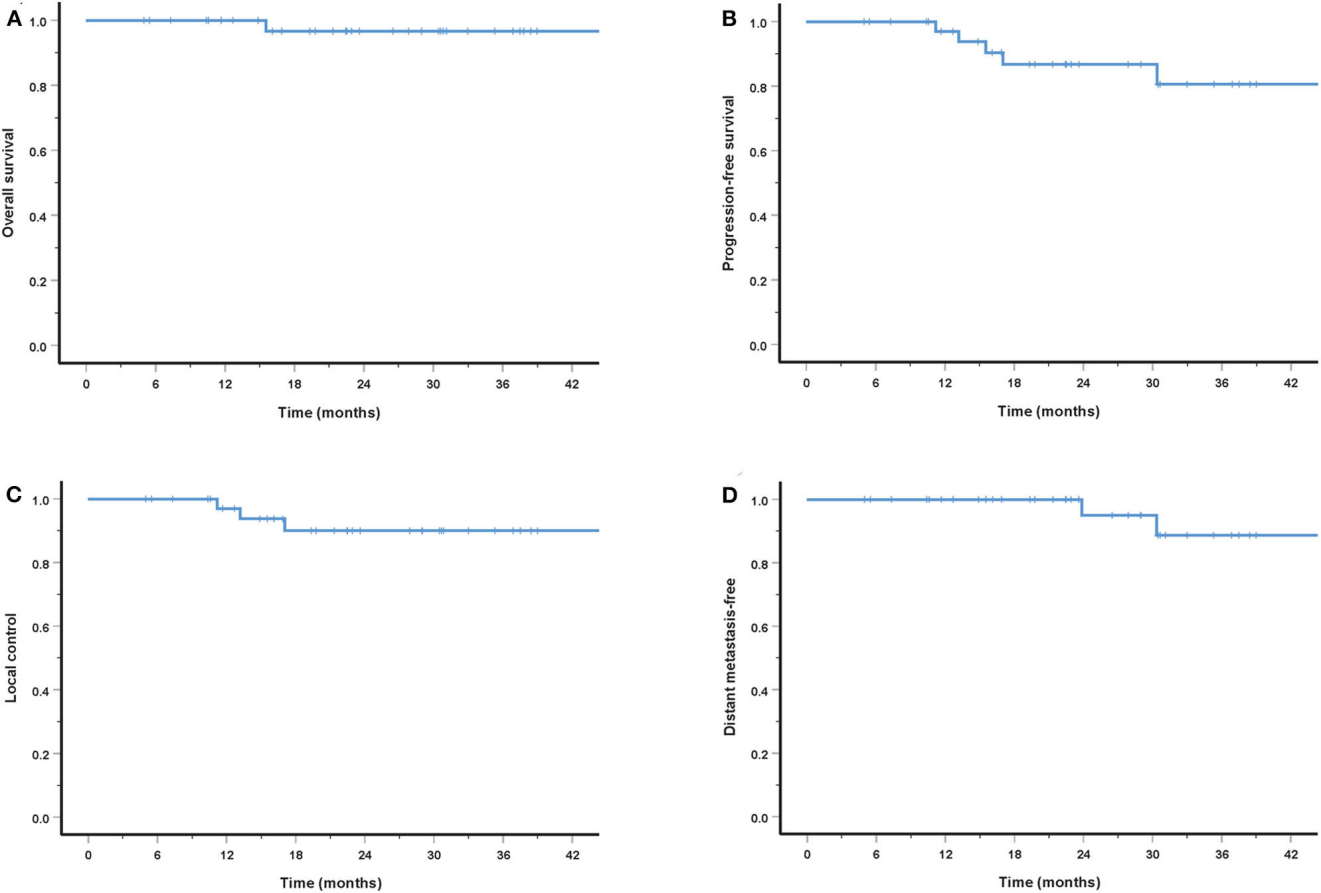
Three-year survival rates for entire cohorts: OS **(A)**, PFS **(B)**, LC **(C)**, and DMF **(D)**.

**Figure 4:**
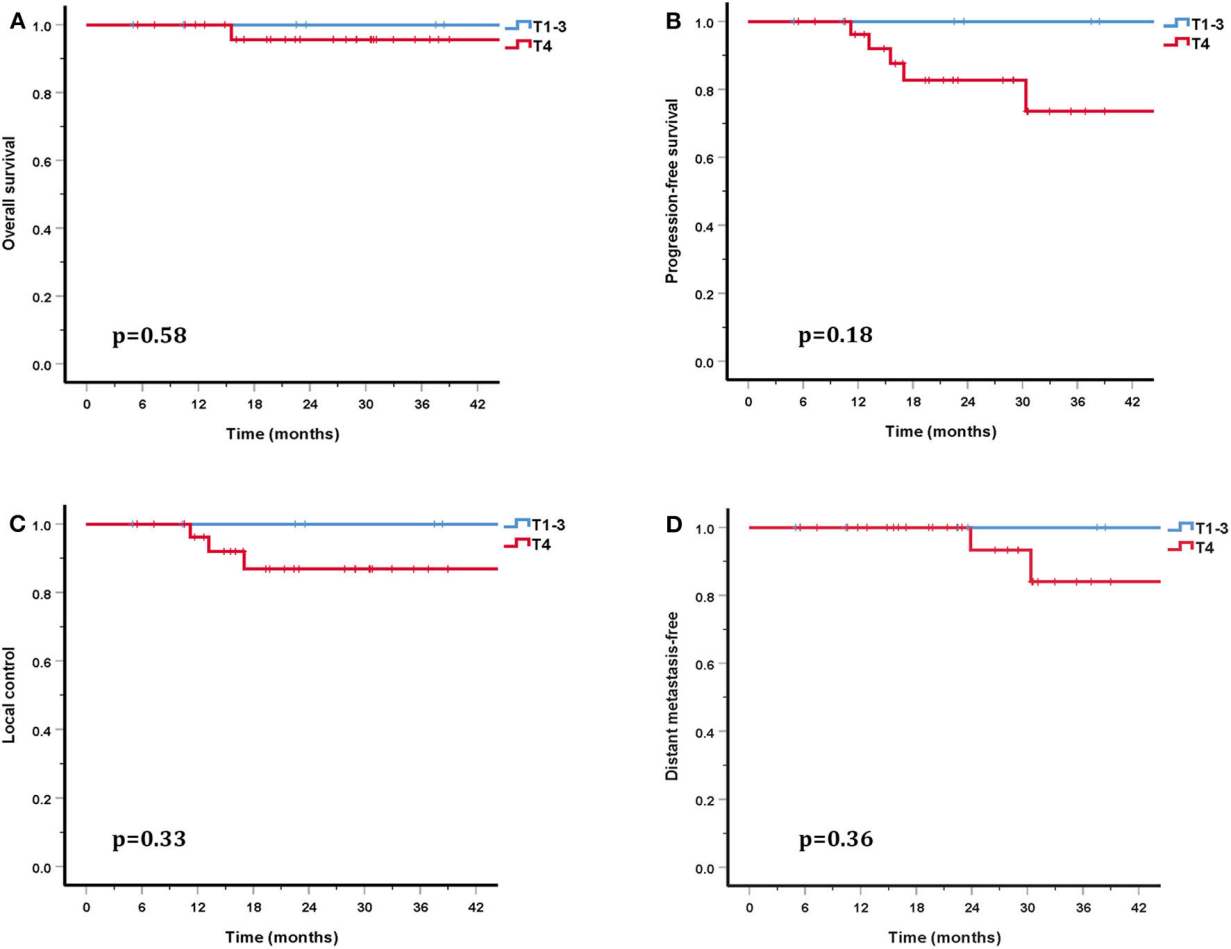
Three-year survival rates for patients with T1-3 disease compared with T4 patients: OS **(A)**, PFS **(B)**, LC **(C)**, and DMF **(D)**.

The cohort's median follow-up was 27.2 months (5–56.6 months). All but one patient were alive at their last follow-up. This patient had received re-irradiation and died from hemorrhage 10 months after the completion of CIRT without evidence of disease progression. Two in-field and one out-field local failure were observed, include two patients who had received CIRT re-irradiation for recurrent disease. Two patients developed DM at 21.5 and 22.8 months after PBRT. No regional failures were observed. The 3-year OS, PFS, LC, RC, and distant metastasis-free (DMF) rates for the entire cohort were 96.7, 80.6, 90.0, 100, and 88.7%, respectively (Figure 3). Although no significant difference was found, the LC and survival rates in patients with T4 disease were poorer (the 3-year OS, PFS, LC, and DMF rates being 95.7, 73.5, 86.9, and 84.0%) compared with T1-3 patients (the 3-year LC and survival rates were all 100%) (Figure 4).

### Toxicities

The acute and late toxicities induced by PBRT are summarized in Table 2. Sixteen patients (42.1%) experienced 19 events of grade 2 acute toxicity, including mucositis, dermatitis, and xerostomia. Ten of the sixteen patients received PBT and CIRT boost treatment, CIRT only was performed in 4 patients and 2 patients only received PBT. No acute toxicities of grade 3 or above were observed.

**Table 2:** Characteristics of acute and late toxicities.

	**Acute toxicities**		**Late toxicities**							
	**Grade 2**		**Grade 1**		**Grade 2**		**Grade 3**		**Grade 5**	
**Type of adverse reaction**	**No**	**%**	**No**	**%**	**No**	**%**	**No**	**%**	**No**	**%**
Dermatitis	1	2.6	0		0		0		0	
Mucositis	15	39.5	0		0		0		0	
Xerostomia	3	7.9	6	15.8	2	5.3	1	2.6	0	
Facial edema	0		1	2.6	0		0		0	
Decreased vision	0		1	2.6	0		1	2.6	0	
Epiphora	0		1	2.6	0		0		0	
Blepharoptosis	0		1	2.6	0		0		0	
Diplopia	0		1	2.6	0		0		0	
Tinnitus	0		1	2.6	0		0		0	
Facial numbness	0		0		1	2.6	0		0	
Hemorrhage	0		0		0		0		1	2.6

Fifteen events of grade 1–2 late toxicity were observed including 9 patients received PBT and CIRT boost radiotherapy, 5 patients were treated with CIRT only and 1 with PBT only. The most common late toxicity was xerostomia. Other grade 1–2 toxicities included facial edema, facial numbness, vision impairment, epiphora, blepharoptosis, diplopia, and tinnitus. Only a small number of grade 3 toxicities occurred, including one event of xerostomia and one of vision impairment. The patient who developed grade 3 vision decreased at three months after the completion of CIRT re-irradiation. One patient died of hemorrhage (grade 5 toxicity associated with re-irradiation) 10 months after completing CIRT re-irradiation as salvage treatment.

## Discussion

The aim of our retrospective study was to describe the outcomes of SNACC patients treated with intensity-modulated PBRT. By examining a cohort of 38 SNACC patients, most of whom had T4 disease (76.3%) or locally recurrent disease after RT (26%), we found that PBT, CIRT, and a combination of the two appeared to be safe and effective. All but 1 patient were alive at a median follow-up of 27.2 months. The 3-year OS, PFS, LC, RC, and DMF rates of the cohort were 96.7, 80.6, 90.0, 100, and 88.7%, respectively. Additionally, no acute grade 3 or above toxicities were observed. Late toxicities of grade 3 or above were observed in 3 patients, including 1 patient who died from hemorrhage 10 months after completing CIRT re-irradiation as salvage treatment.

SNACC is a rare condition with poor prognosis that accounts for 10–25% of all head and neck ACC (11, 12). Given the complicated anatomy of the sinonasal region, gross tumor resection is difficult to achieve and can result in LC failure (13, 14). SNACC is a chemotherapy-resistant condition (15–22). Postoperative RT is usually used to improve LC and survival in these patients. The results of several retrospective studies have shown that, compared with surgery alone, the combination of RT and surgery not only decreases local failures, but also improves 5-year disease-specific survival (DSS) (4, 6). However, for patients with unresectable tumors, definitive radiotherapy is the only approach. As a result of it radioresistant nature (5), several recent researches reported an inferior 5-year LC of 42–56% in head and neck ACC (23, 24), with the 5-year DSS rate being only 37.3% for SNACC patients treated with RT alone (6).

Due to this radioresistance, PBRT, including neutron therapy, PBT, and CIRT, has been used to improve disease control rates. A prospective randomized phase-III trial comparing the LC rate of neutron and photon radiotherapy (13) found a 10-year LC rate of 56% in neutron therapy compared with 17% in conventional RT. Another retrospective study showed a significantly higher 5-year LC rate of 75% for the neutron therapy group compared with 32% in the photon therapy group (14). However, severe toxicities were more prevalent in the neutron therapy group (19%) than in the photon radiotherapy group (4%) (13, 14).

Due to the Bragg peak, PBT and CIRT can provide a more precise dose distribution, facilitating high-dose irradiation of tumor volume while sparing OARs. In a case series of 13 patients with locally advanced SNACC, Dautruche et al. retrospectively analyzed the survival of patients treated with PBT and/or tomotherapy. They reported 3-year OS, LC and DM free survival (DMFS) rates of 60, 48, and 60%, respectively (25). Recently, a retrospective study of postoperative PBT for head and neck ACC showed promising LC (26): 94% of patients achieved R0 or R1 resection with a median follow-up of 24.9 months. Only one patient developed in-field recurrence. Another study by Pommier et al. analyzed the efficacy of PBT combined with photon radiotherapy in the treatment of 23 ACC patients with skull base invasion. They reported 5-year LC and OS rates of 93% and 77% (27). More recently, Linton et al. reported 2-year LC and OS rates of 92 and 82% in head and neck ACC patients treated with PBT (28). Although a higher LC rate was achieved in PBT, the incidence of radio-induced toxicities was still high. The incidence rates of severe acute and late toxicities were 6.3–26 and 4–13%, respectively (25–28).

In addition to PBT, CIRT is a high LET beam and has a higher RBE compared with proton and photon beams. In the COSMIC study, Jensen et al. investigated the efficacy and safety of IMRT combined with a CIRT boost for salivary malignancies. With a median follow-up of 42 months, the 3-year LC in ACC patients was 81.9% (29). In another study by the same researchers, Jensen et al. also compared the outcomes of IMRT and IMRT combined with a CIRT boost (30). This study included 95 inoperable or subtotal resection head and neck ACC patients. This study showed that the LC and OS rates were significantly higher in the CIRT boost group than in photon group (the 10-year LC and OS rates were 42.2 and 44.2% in the CIRT boost group and 32 and 19.6% in the IMRT alone group) (30). Another study included 309 head and neck ACC patients treated with IMRT and a CIRT boost, of whom 61% had T4 disease. The overall 3-year LC and OS rates were 83.7 and 88.9%, respectively (31). However, the 3-year LC and OS rates for T4 patients were 75.9–89.6% and 38.6–72.5%. This study also revealed that T classifications were significant prognostic factors for LC (31). More recently, a retrospective study that included 227 SNACC patients treated with IMRT combined with a CIRT boost reported 3-year LC and OS rates of 82 and 79% for patients who had received surgery before PBRT, and 79 and 64% for patients who did not receive surgery (32). This study also showed that patients with T4 disease had a significantly poorer LC rate (32). A retrospective study of CIRT alone with the largest number of head and neck ACC patients analyzed 289 eligible patients, including 122 with SNACC, reported 2-year OS and PFS rates of 94 and 68% (8). In this study, 41 patients (15%) had local recurrence. Both multivariate and univariate analysis suggested that a large GTV and T4 disease were associated with lower LC, OS, and PFS (8). In our study, 76.3% of patients suffered T4 disease and gross tumor was present in 84.2% patients. Although no significant difference was found, the 3-year LC and OS rates of patients with T4 disease (86.9 and 95.7%) were lower than that of T1-3 patients (LC and OS rates were both 100%). These observations were consistent with previous research. While there were many adverse prognostic factors present in these patients, our study still produced promising outcomes, the 3-year OS, PFS, LC, and DMF rates being 96.7, 80.6, 90.0, and 88.7%.

PBRT provides precise dose distribution to tumor volumes while sparing OARs. Studies report acute grade 2 toxicities, such as dermatitis, mucositis, and dysphagia occur in 27–62% of patients and the incidence rate of acute grade 3 dermatitis or mucositis was 6.3–26% after PBT (25–28). PBT-induced severe (grade 3 or 4) late toxicities (osteoradionecrosis, otologic, or ocular toxicities) occur in 4–13% of patients (27, 28). However, the incidence rate of brain injury induced by PBT in patients with skull base invasion is as high as 56.5%. Of these patients, 43.5% had a grade 3 brain injury (27). In a study using bimodal treatment (a combination of IMRT and CIRT), German researchers reported that the incidence rate of acute grade 3 toxicities, such as dermatitis, mucositis, dysphagia, or conjunctivitis, was between 4 and 42%, and severe late toxicities were reported in 2–17% of patients (29–32). The incidence rates of central neural system necrosis or cranial paralysis were reported at 2–6% (30, 31). In the above-mentioned study of CIRT alone for head and neck ACC treatment, Sulaiman et al. described acute grade 3 mucositis, grade 3 dermatitis, and late grade 3 or above toxicities (including osteoradionecrosis, visual impairment, brain injury, hemorrhage, and mucositis) occurring in 29, 3.8, and 15% of patients, respectively (8). In our study, no patients suffered acute toxicities of grade 3 or above. The incidence rates of grade 2 dermatitis, mucositis, and xerostomia were 2.6, 39.5, and 7.9%, respectively. Severe late toxicities occurred in three patients. One patient with orbital invasion who received CIRT re-irradiation developed grade 3 vision impairment. Another patient suffered grade 3 xerostomia. One patient with recurrent T4 maxillary sinus ACC developed grade 5 hemorrhage at 10 months after completing CIRT re-irradiation.

As head and neck ACC is insensitive to systemic therapy, distant metastasis remains a major problem; recent studies have found that the distant failure rate ranges between 30 and 55% (8, 25–32). Moreover, previous research has shown a significant linear correlation between distant metastasis and OS. In our cohort, the DM rate was 5.3%; however, this incidence rate may have been underestimated due to insufficient follow-up time. We also failed to find a correlation between DM and survival rates, which may be attributable to the short follow-up time and our small sample size. The latest ACCEPT trial found that the combination of cetuximab and IMRT with a CIRT boost was a feasible approach for head and neck ACC treatment. Long-term follow-up to verify the benefit in survivals is underway (33).

Two limitations of this study need to be discussed, the major one being its retrospective nature. However, due to the rarity of SNACC, this disease is predominantly investigated in retrospective studies, case reports, or single-center experiences. Secondly, we had tried to use COX regression model or competing risk model, but the sample size and number of events were too small for prognostic factor analyses. Additionally, as SNACC is a slow-growing tumor, the median follow-up time of 27.2 months was relatively short. Therefore, a longer follow-up time is needed.

## Conclusion

Our results showed that PBRT is an effective and safe treatment option for SNACC. The 3-year OS, PFS, LC, RC, and DMC rates were 96.7, 80.6, 90.0, 100, and 88.7%, respectively. The toxicities related to PBRT were infrequent and mild. No severe acute toxicities were observed, but three patients developed severe late toxicities. Longer follow-up is needed to verify the long-term benefit of PBRT for patients with SNACC.

## Data Availability Statement

The raw data supporting the conclusions of this article will be made available by the authors, without undue reservation.

## Ethics Statement

The studies involving human participants were reviewed and approved by institutional review board (IRB) of Shanghai Proton and Heavy Ion Center with a waiver of informed consent (IRB No. 200220EXP-02).

## Author Contributions

LK, JL, and WH: conception, design and final approval. WH, JH, QH, XQ, and JY: acquisition and assembly of data. WH, JH, and JG: date analysis and interpretation. WH, JH, LK, and JL: drafting or revising the article. LK: funding acquisition. All authors contributed to the article and approved the submitted version.

## Conflict of Interest

The authors declare that the research was conducted in the absence of any commercial or financial relationships that could be construed as a potential conflict of interest.
